# Effect of Educational Outreach Timing and Duration on Facility Performance for Infectious Disease Care in Uganda: A Trial with Pre-Post and Cluster Randomized Controlled Components

**DOI:** 10.1371/journal.pone.0136966

**Published:** 2015-09-09

**Authors:** Sarah M. Burnett, Martin K. Mbonye, Sarah Naikoba, Stella Zawedde-Muyanja, Stephen N. Kinoti, Allan Ronald, Timothy Rubashembusya, Kelly S. Willis, Robert Colebunders, Yukari C. Manabe, Marcia R. Weaver

**Affiliations:** 1 Accordia Global Health Foundation, Washington, District of Columbia, United States of America; 2 Infectious Diseases Institute, College of Health Sciences, Makerere University, Kampala, Uganda; 3 Center for Human Services, University Research Co. LLC, Bethesda, MD, United States of America; 4 Department of Medicine, University of Manitoba, Winnipeg, Manitoba, Canada; 5 Department of Epidemiology and Social Medicine, Faculty of Medicine and Health Sciences, University of Antwerp, Antwerp, Belgium; 6 Department of Clinical Sciences, Institute of Tropical Medicine, Antwerp, Belgium; 7 Division of Infectious Diseases, Department of Medicine, Johns Hopkins University School of Medicine, Baltimore, Maryland, United States of America; 8 International Training and Education Center for Health (I-TECH), Department of Global Health, University of Washington, Seattle, Washington, United States of America; University of Ottawa, CANADA

## Abstract

**Background:**

Classroom-based learning is often insufficient to ensure high quality care and application of health care guidelines. Educational outreach is garnering attention as a supplemental method to enhance health care worker capacity, yet there is little information about the timing and duration required to improve facility performance. We sought to evaluate the effects of an infectious disease training program followed by either immediate or delayed on-site support (OSS), an educational outreach approach, on nine facility performance indicators for emergency triage, assessment, and treatment; malaria; and pneumonia. We also compared the effects of nine monthly OSS visits to extended OSS, with three additional visits over six months.

**Methods:**

This study was conducted at 36 health facilities in Uganda, covering 1,275,960 outpatient visits over 23 months. From April 2010 to December 2010, 36 sites received infectious disease training; 18 randomly selected sites in arm A received nine monthly OSS visits (immediate OSS) and 18 sites in arm B did not. From March 2011 to September 2011, arm A sites received three additional visits every two months (extended OSS), while the arm B sites received eight monthly OSS visits (delayed OSS). We compared the combined effect of training and delayed OSS to training followed by immediate OSS to determine the effect of delaying OSS implementation by nine months. We also compared facility performance in arm A during the extended OSS to immediate OSS to examine the effect of additional, less frequent OSS.

**Results:**

Delayed OSS, when combined with training, was associated with significant pre/post improvements in four indicators: outpatients triaged (44% vs. 87%, aRR = 1.54, 99% CI = 1.11, 2.15); emergency and priority patients admitted, detained, or referred (16% vs. 31%, aRR = 1.74, 99% CI = 1.10, 2.75); patients with a negative malaria test result prescribed an antimalarial (53% vs. 34%, aRR = 0.67, 99% CI = 0.55, 0.82); and pneumonia suspects assessed for pneumonia (6% vs. 27%, aRR = 2.97, 99% CI = 1.44, 6.17). Differences between the delayed OSS and immediate OSS arms were not statistically significant for any of the nine indicators (all adjusted relative RR (aRRR) between 0.76–1.44, all p>0.06). Extended OSS was associated with significant improvement in two indicators (outpatients triaged: aRR = 1.09, 99% CI = 1.01; emergency and priority patients admitted, detained, or referred: aRR = 1.22, 99% CI = 1.01, 1.38) and decline in one (pneumonia suspects assessed for pneumonia: aRR: 0.93; 99% CI = 0.88, 0.98).

**Conclusions:**

Educational outreach held up to nine months after training had similar effects on facility performance as educational outreach started within one month post-training. Six months of bi-monthly educational outreach maintained facility performance gains, but incremental improvements were heterogeneous.

## Introduction

With 24% of the global burden of disease and only three percent of the world’s health workers, the shortage of healthcare workers (HCWs) in sub-Saharan Africa is a major barrier to meeting the Millennium Development Goals [1]. At the same time, these existing HCWs require on-going capacity development to continuously update their knowledge and skills to align with changes in national health policies and treatment guidelines.

Several studies have demonstrated that didactic, classroom-based, in-service trainings, a common form of capacity development throughout the world, are not sufficient to ensure adherence to clinical guidelines [2–4]. These trainings also take HCWs away from busy health facilities, leaving these facilities even further understaffed. A recent study in Uganda found that 15% of HCWs were absent from their facility due to trainings [5]. Capacity building methods that allow HCWs to remain on-site in understaffed health facilities may be particularly suited for low-resource settings.

Donors and organizations implementing capacity development programs have shown increasing interest in educational outreach and continuous quality improvement as key methods for building HCWs’ capacity and improving the quality of care [6–10]. Educational outreach is described as “a personal visit by a trained person to health professionals in their own settings” [11]. Continuous quality improvement is made up of three essential features: systematic data guided activities, designing with local conditions in mind, and iterative development and testing [12]. In addition to reduced time away from health facilities, these interventions offer staff development activities that are directly relevant to HCWs’ work environment and provide increased opportunities for team-based interaction [6].

Recent randomized control trials and reviews reveal that educational outreach, which sometimes include continuous quality improvement activities, can improve the quality of patient care [11,13–18]. However, less is known about the required timing, duration and frequency of such interventions [11,19]. In a cluster randomized control trial of the World Health Organization’s Integrated Management of Childhood Illnesses (IMCI) program in Benin, HCWs and those who received on-going supervisory visits (with two visits every three months as the recommended frequency) maintained a higher level of performance, even though only 29% of planned visits occurred [20] compared to those who received one supervisory visit one month after IMCI training. Both groups maintained their performance on three quality of care indicators three years after the initial training [21].

Our study adds new information about the effect of timing, duration, and frequency of educational outreach activities on facility performance. This article presents the results from Phase 2 of the Integrated Infectious Disease Capacity Building Evaluation (IDCAP) [6,13]. In Phase 1, IDCAP conducted a cluster randomized control trial to test the effect of the Integrated Management of Infectious Disease (IMID) training program based at the Infectious Diseases Institute in Kampala and on-site support (OSS), an educational outreach intervention with continuous quality improvement activities, on individual clinician competence [22] and practice, facility performance [13,23] and population-based mortality of children less than five years [(Naikoba, etal., 2012)]. The two objectives of this Phase 2 analysis were to: a) test the combined effect of training and OSS when they are given sequentially compared with a nine month delay and b) test the effect of extended OSS on facility performance. We examined indicators in three program areas with pre/post improvements in Phase 1. Results from the arm with delayed implementation provide an opportunity to examine the effects of timing of the interventions. When reporting the phase 1 trial results for the performance indicators [13], we recommended continuing OSS over longer time period and concentrating effort on specific indicators. The results from phase 2 are an opportunity to examine the effects of continued OSS.

## Methods

### Study Design

**Fig 1:**
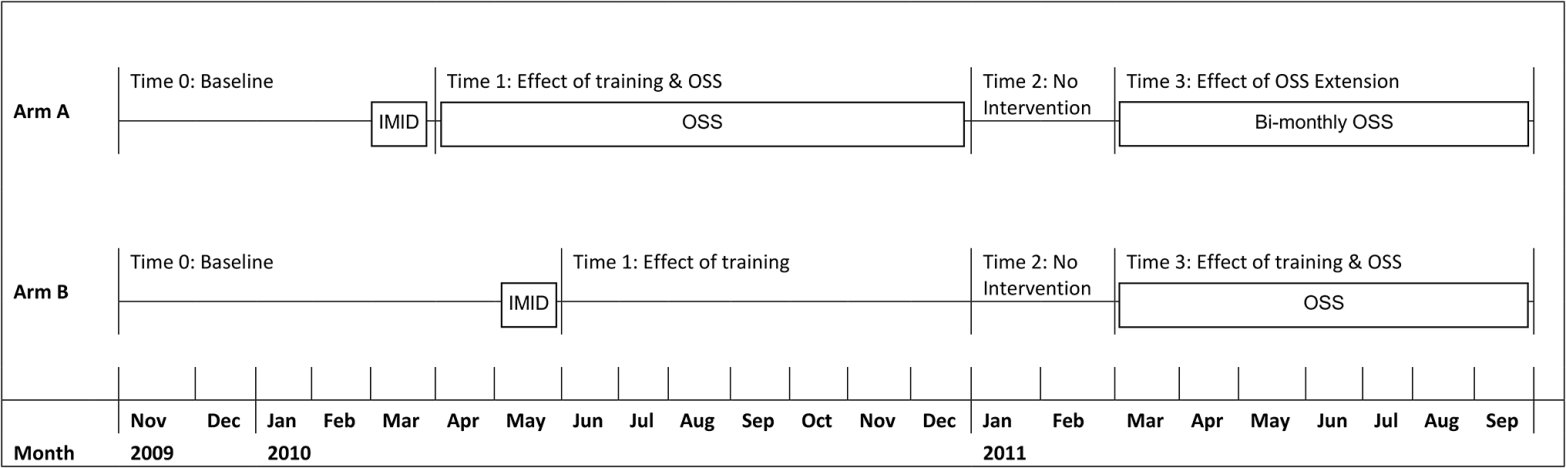
Evaluation Design.

This study was conducted at 36 health centers IV or comparable facilities in Uganda. Each health facility acted as a cluster and was randomized (1:1) to parallel arms. Health facility data were collected prospectively from November 2009 to September 2011. The two time periods in Phase 1 were baseline (time 0) and randomized trial (time 1) of OSS (Fig 1). Time 0 started November 2009 and ended in March 2010 for arm A and May 2010 for arm B. Two mid-level practitioners at each of the 36 sites attended the IMID training program beginning in March 2010. In time 1 18 sites in arm A received nine monthly OSS visits from April 2010 to December 2010, and 18 sites in arm B did not. The two time periods in Phase 2 were a brief period with no intervention (time 2), January and February 2011, and the delayed intervention period (time 3), March to September 2011. In time 3, arm A received three additional OSS visits every two months (extended OSS), while arm B sites received the eight monthly OSS visits, nine months after their IMID training (Fig 1).

The effect of timing of OSS was tested by comparing the combined effect of training and delayed OSS on arm B (time 3 vs. time 0) to the combined effect of training and immediate OSS on arm A (time 1 vs. time 0). The effect of the duration of OSS was tested by comparing arm A during extended OSS (time 3) to the same facilities during training and immediate OSS (time 1).

The study design is summarized in Naikoba et al. [6] and the study protocol can also be accessed as Protocol S1 in Weaver et al. [13] and Mbonye et al. [24]. The CONSORT checklist for this trial is in S1 Table. Anonymous data for the analyses reported below are available for public use on the Global Health Data Exchange website http://ghdx.healthdata.org/record/uganda-integrated-infectious-disease-capacity-building-evaluation-facility-level-data-2009.

#### Participants and eligibility

Two mid-level practitioners (MLP), consisting of clinical officers, registered nurses, or registered midwives from each of the 36 health centers IV or comparable small hospitals in Uganda participated in the IMID training program. Health centers IV act as an initial referral facilities with limited inpatient wards for the health subdistricts within Uganda and provide basic preventative and curative care and referral services for health sub-districts’ populations of about 100,000 [25]. Inclusion criteria for facilities and IMID participants have been described previously [6]. All facility staff were invited to participate in the OSS intervention. All outpatients at the facilities participated as part of their routine process of care.

#### Interventions

The Integrated Management of Infectious Disease (IMID) training program included a three-week core course, two one-week boost courses at 12 and 24 weeks after the core course and distance learning as described in Miceli et al. [26]. The course was taught at the Infectious Diseases Institute in Kampala. As described in Naikoba et al., OSS visits were two-day visits by a four-person mobile team: a medical officer, clinical officer, laboratory technologist, and registered nurse [6]. During time 1, OSS visits were conducted once a month for nine consecutive months in arm A [6]. During time 3, arm A received an OSS visit every other month, for a total of three additional OSS visits, while arm B received an OSS visit every month for a total of eight visits. Over each two-day visit, the mobile team conducted four activities: multidisciplinary team training, one-on-one mentoring, break-out sessions by cadre, and continuous quality improvement. Each visit would focus on a specific topic based on the training program materials, as well as follow-up on topics from previous visits. Although the topics presented were the same in both phases, the sequence in time 1 was reported in Naikoba et al., and the sequence in time 3 in Miceli et al. [6,26]. During the OSS extension period for arm A the three sessions focused on pediatric ART, TB case management, and fever and malaria case management.

#### Outcomes

The outcome measures were nine facility performance indicators across three of four areas that showed improvement in time 1: emergency triage, assessment, and treatment; malaria; and pneumonia [13] (Table 1). The fourth area of care, enrollment in HIV care, will be addressed in a separate manuscript.

**Table 1:** Definitions and data sources for facility performance indicators.

Program Area and Performance Indicators		Definition
**Emergency Triage, Assessment, and Treatment (ETAT)**		
*Definitions presented in Kinoti et al*., *unpublished manuscript*		
1	Proportion of outpatients triaged	**Numerator:** Number of outpatients triaged, meaning that the patient was classified as emergency (ABCDO triage categories: Airway; Breathing difficulty; Circulation / Coma / Convulsion / Confusion; Dehydration; and Other), priority (3TPR-MOB priority signs: Tiny baby (sick child of less than 2 months of age); Temperature (child is very hot); Trauma or other urgent surgical condition; Pallor (severe); Poisoning; Pain (severe); Respiratory distress; Restless (lethargy or continuously irritable); Referral; Malnutrition (severe wasting); Oedema of both feet; and Burns), or queue, or an emergency sign was noted in the triage section of the form. **Denominator:** Number of outpatients
2	Proportion of emergency and priority patients who were admitted, detained or referred	**Numerator:** Number of emergency and priority patients admitted, detained or referred for care. **Denominator:** Number of outpatients classified as emergency or priority or an emergency sign was noted in the triage section of the form
3	Estimated proportion of emergency patients who received at least one appropriate treatment	**Numerator:** Number of emergency patients who received at least one emergency drug. The drugs that could be used for emergency care and that were listed in the revised Medical Form 5 were artesunate, aspirin, benzyl penicillin (X-pen), chloramphenicol, cloxacillin, diazepam, gentamycin, intravenous fluids, magnesium, oxygen, oral rehydration solution, phenytoin, quinine, and salbutamol. Use of the following eight “other” drugs also met the criteria for appropriate treatment: ampicillin, benzathine penicillin, ceftriaxone, cefuroxime, epinephrine, paraldehyde, pencillin (generic), and phenoxymethyl penicillin. For emergency patients who were prescribed treatment and data on drug availability were missing, we applied the “in-stock” rate for patients with those data. **Denominator:** Number of outpatients classified as emergency or an emergency sign was noted in the triage section of the form
**Case management of fever and malaria**		
*Definitions presented in Mbonye et al*. *[23]*		
4	Proportion of malaria suspects with a malaria test result recorded	**Numerator:** Number of malaria suspects with a result for a laboratory test or rapid diagnostic test for malaria, where the definition of a malaria suspect was all patients with a fever, referred for malaria laboratory testing, or given a clinical diagnosis of malaria as evidenced by either a record of malaria diagnosis or an antimalarial prescription. **Denominator:** Number of malaria suspects
5	Estimated proportion of malaria cases who received an appropriate antimalarial	**Numerator**: Number of outpatients treated with appropriate anti-malarial(s), where appropriate antimalarial treatments were quinine or artesunate and the following ACTs: artemether & lumenfantrine, artesunate & amodiaquine, or dihydroartemisinin & piperaquine phosphate (Duocotecxin). For patients who were prescribed an antimalarial and data on drug availability were missing, we applied the “in-stock” rate for patients with those data. **Denominator**: Number of outpatients treated for malaria
6	Proportion of patients with a negative malaria test result who were prescribed an antimalarial	**Numerator:** Number of patients with a negative malaria test result prescribed any antimalarial including the appropriate antimalarials listed above and three drugs that did not comply with Uganda national guidelines: amodiaquine alone, chloroquine, and sulfadoxine-pyrimethamine. **Denominator:** Number of patients with a negative malaria test result
7	Proportion of patients with a positive malaria test result who were prescribed an antibiotic	**Numerator**: Number of patients with a positive malaria test result prescribed any antibiotic(s). Any antibiotic treatment referred to 12 drugs listed on the MF5: amoxicillin, benzyl penicillin, chloramphenicol, ciprofloxacin, cloxacillin, cotrimoxazole, doxycycline, erythromycin, gentamicin, metronidazole, PPF/procaine penicillin, tetracycline. Data on these drugs was elicited by checking boxes on the MF5. It also included 19 antibiotics recorded as ‘‘other drugs:” Ampiclox (ampicilllin & cloxacillin), ampicillin, ampicillin & gentamicin, azithromycin, cefalexin, cefixime, ceftriaxone, cefuroxime, co-amoxiclav, dapsone, dicloxacillin, gatifloxacin, levofloxacin, nalidixic acid, nitrofurantoin, ofloxacin, pencillin (generic), perfloxacin, phenoxymethyl penicillin. **Denominator:** Number of patients with a positive malaria test result
**Case management of respiratory illness**		
*Definitions presented in Weaver et al*. *[13]*		
8	Proportion of pneumonia suspects aged under 5 years assessed for pneumonia	**Numerator:** Number of child pneumonia suspects with at least one of the three following assessment results recorded: 1) abnormal chest sounds, 2) chest in-drawing, and 3) rapid breaths per minute. A pneumonia suspect was defined as any child aged under five years presenting with cough or who received a diagnosis of “pneumonia” or “cough/cold no pneumonia”**. Denominator:** Number of child pneumonia suspects. **Note**: The definition of suspect focused on children with cough; difficulty in breathing was inadvertently omitted from the form.
9	Estimated proportion of patients aged under 5 years diagnosed with pneumonia who received appropriate antibiotic treatment	**Numerator:** Number of children diagnosed with pneumonia treated with appropriate antibiotic, where appropriate antibiotic treatment referred to six drugs on the revised Medical Form 5: amoxicillin, benzyl penicillin, erythromycin, chloramphenicol, gentamicin, cotrimoxazole, and 11 other drugs that were specified: ampicillin, azithromycin, cefixime, ceftriaxone, cefuroxime, co-amoxiclav, gatifloxacin, levofloxacin, penicillin, phenoxymethylpenicillin, ampiclox (amoxicillin and cloxacillin). For patients who were prescribed an antibiotic and data on drug availability were missing, we applied the “in-stock” rate for patients with those data. **Denominator:** Number of children diagnosed with pneumonia

#### Variable definitions and data sources

Definitions of the nine indicators are presented in Table 1 as originally reported in Weaver et al. [13] and Mbonye et al. [24]. All nine indicators used a modified version of the Ministry of Health’s Medical Form 5 (MF5), an outpatient record [6,24].

#### Sample size

Sample size calculations were included in the protocol and reported in Naikoba et al. [6]. Data from Ssekabira et al. were used to calculate the sample size required to detect an effect of OSS on facility performance on two malaria indicators with the facility as the unit of analysis: 1) the percentage of malaria suspects with a malaria test recorded, which had a baseline of 38% among children less than five years and increased by 16%, and 2) the percentage of patients with a negative malaria test result who were prescribed an antimalarial, which had a baseline of 48% among children less than five years and decreased by 16% [27]. Using the health facility as the unit of analysis, we calculated the number of facilities required to detect a 20% absolute difference between the intervention and control arms with a power of 80% and an alpha of 0.05.

#### Randomization

The identification and selection of sites was conducted by the investigators and project staff. The 36 facilities selected to participate in the study were stratified to control for two other interventions: 1) previous participation in a national HIV continuous quality improvement program and 2) the on-going Baylor International Pediatric AIDS Initiative [28,29]. Within these strata, the 36 sites were randomized to parallel arms (1:1 balance). On February 23, 2010, the IDCAP biostatistician randomized the 36 facilities. A random number generator in Stata was used to assign sites to the two arms–with the mean of the generated random numbers acting as a cut-off point. Numbers less than the mean were allocated to arm A and those above the mean were allocated to arm B [6,23]. The project staff and health workers at the facilities were blinded during four months of baseline data collection, but not blinded during the intervention.

#### Ethical Considerations

The School of Medicine Research and Ethics Committee of Makerere University Kampala, Uganda (reference number 2009–175) and the Uganda National Council for Science and Technology (reference number HS-722) reviewed and approved the IDCAP protocol. The University of Washington Human Subjects Division determined that IDCAP did not meet the regulatory definition of research under 45 CFR 46.102(d). Participants in the infectious disease training course provided written informed consent. OSS participants were not asked to provide informed consent for facility performance data, because the facility performance data were used to evaluate facility rather than individual performance. Informed consent of patients was waived for the MF5 data.

#### Data Collection

Data were collected on every outpatient visit from November 2009 to September 2011 using the revised MF5 forms, which were completed by records staff, clinicians, lab personnel and drug dispensers. Beginning in March 2010, data entry assistants stationed at each facility entered the revised MF5 data in an Epi Info database (Version 3.2, U.S. Centers for Disease Control and Prevention, Atlanta, GA). The data entry assistants electronically transmitted the revised MF5 data to the Infectious Disease Institute on a monthly basis, where the data were merged using Microsoft Excel (Microsoft Corporation, Redmond, WA, USA), cleaned, and exported to Stata version 11 (StataCorp, College Station, Texas, USA) for analysis.

#### Data analysis

Data collected from November 2009 to September 2011 were analyzed, and the facility-month was the unit of analysis. The Phase 1 time periods of time 0 (baseline) and time 1 (intervention) remain the same as reported in Weaver et al. and differed by arm [13] (Fig 1). For arm A, time 0 was from November 2009 to March 2010 and time 1 was from April 2010 to December 2010. For arm B, time 0 was from November 2009 to May 2010 and time 1 was from June 2010 to December 2010. In Phase 2, two additional time periods covered the same months in each arm. The period after OSS ended in arm A and before OSS started in arm B, time 2, was from January 2011 to February 2011. Delayed OSS in arm B and OSS extension in arm A, time 3, was from March 2011 to September 2011.

To determine the effect of the timing of OSS, we compared the combined effect of training and immediate OSS on arm A during OSS implementation (time 1 vs. time 0) to training and delayed OSS on arm B during OSS implementation (time 3 vs. time 0). To determine whether facility performance was maintained, improved, or declined during the OSS extension period in arm A, we compared the extended OSS period (time 3) to the period of training and OSS implementation (time 1).

As described in Weaver et al. and Mbonye et al., the data were analyzed using the generalized linear model with a Poisson family and log link to estimate the relative risks (RR) for the proportion of patients managed appropriately for a given indicator with main effects for arm, time period, and their interaction [13,24]. The unit of analysis was the facility month.

All regression analyses were clustered by facility to adjust for random facility effects and used robust standard errors to adjust for using the Poisson instead of the binomial family and for overdispersion. To address the multiple comparisons, tests were based on a one percent level of significance, and the results are presented with 99% confidence intervals (CI). All analyses adjusted for facility type, facility level, data entry assistant stationed at the site, staffing and previous participation in the national HIV continuous quality improvement program and the on-going Baylor International Pediatric AIDS Initiative, as described in Weaver et al. [13]. All analyses were performed with Stata version 11 (StataCorp, College Station, TX, USA).

## Results

### Participant Flow

**Fig 2:**
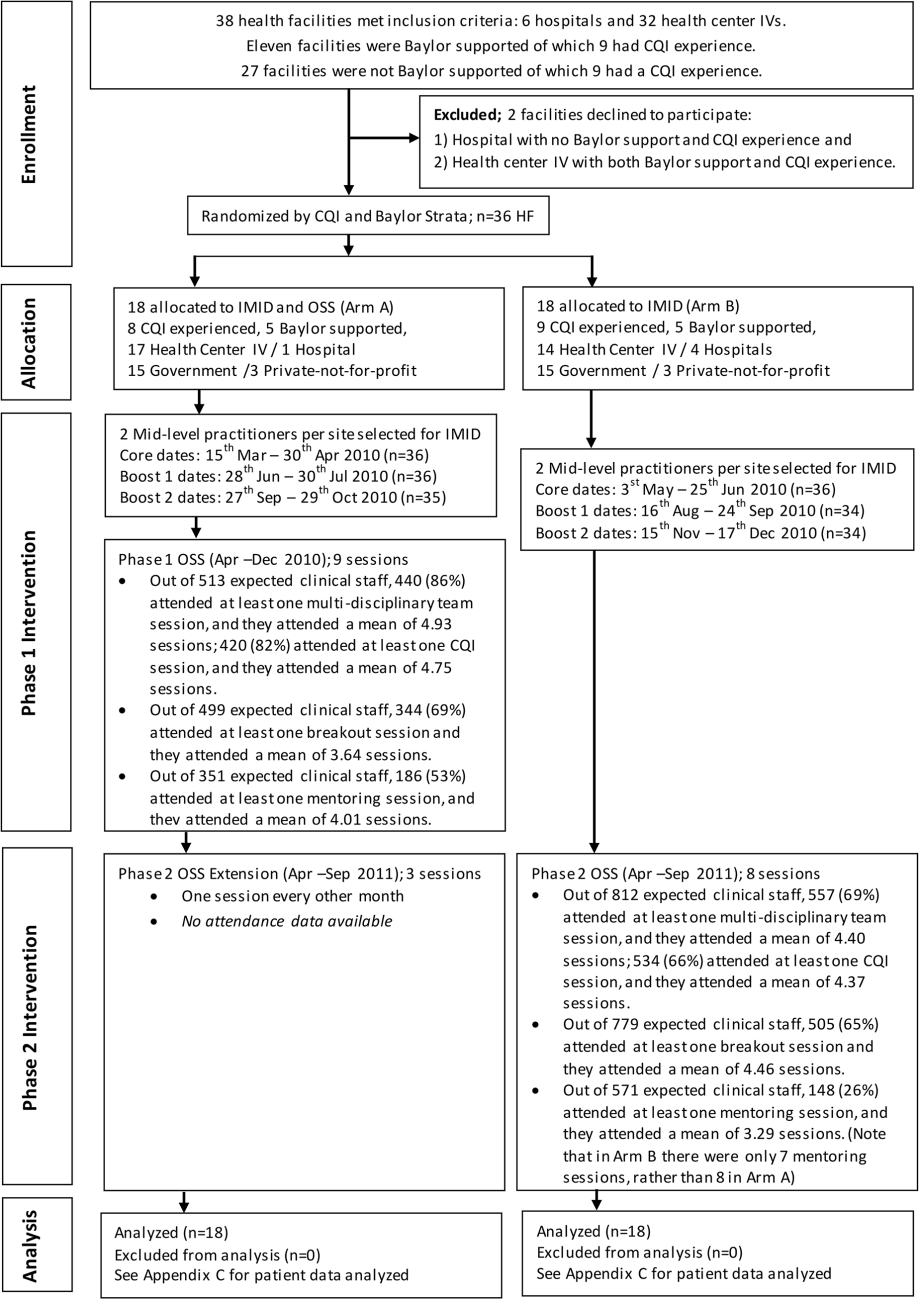
Recruitment and Participation Flow Chart.

The flow of facilities and individuals in infectious disease training program and OSS is shown in Fig 2. Of the 36 health facilities, 31 were health centers IV and five were hospitals. Four of the five hospitals were randomly assigned to arm B. There was no attrition among the 36 enrolled facilities. Participation in Phase 1 of the study is reported in Weaver et al. [13]. Among the 72 training participants, three in arm A and four in arm B discontinued seeing patients in the outpatient department during the course of the study. Arm B in Phase 2 had lower OSS attendance than arm A in Phase 1. Only 557 out of 812 (69%) clinical staff attended at least one multi-disciplinary team training session during OSS, compared to 86% in arm A Phase 1. This difference was primarily due to the hospitals in arm B. In arm A, the hospital staff accounted for 10% of the 513 clinical staff expected to attend OSS, whereas in arm B, hospital staff accounted for 45% of the 812 expected clinical staff. Comparing attendance at multi-disciplinary team training sessions in only health centers IV, both arms had an 80% attendance rate. The four hospitals in arm B had a lower attendance rate (55%), whereas the one hospital in arm A had a 90% attendance rate. Lower attendance among hospitals in arm B could be explained by staff assignments to evening and night shifts which would lead them to being off-duty during some of the OSS sessions, as well as differences in management across hospitals. Among the four hospitals in arm B attendances rates varied from 31% to 81% of the expected facility staff. Attendance data were not available for the OSS extension period in arm A.

A total of 1,275,960 outpatients were seen in the 36 facilities over 23 months, from November 2009 to September 2011. For all indicators the response rate was over 99% for the expected facility months. The total number of patients used in each of the analyses is presented in S1 Fig For one emergency triage, assessment, and treatment indicator (outpatients triaged), a total of 1,656 facility month observations (36 facilities over 23 months broken into two age groups) were expected. For the two pneumonia indicators, which focused on children under five, only 828 observations were expected. For the rest of the indicators the number of facility months available for analysis depended upon whether any patients meeting the denominator definition were seen that month. For example, 237 facility months had no emergency patients identified, thus there were only 1,413 observations possible for the estimated proportion of emergency patients who received at least one appropriate treatment indicator.

### Recruitment

The facilities were recruited between March and September 2009. The IMID participants were recruited between June 2009 and February 2010. Registration and consent for the training was carried out between December 2009 and March 2010. OSS recruitment and registration began in April 2010 and continued throughout the Phase 1 and Phase 2 interventions. All staff were encouraged to attend OSS sessions regardless of previous attendance.

### Baseline

Baseline data on each indicator by arm were reported in Weaver et al. [13] and are shown in S1 Fig. Baseline performance for all nine indicators was below 60%. For two of the indicators, a lower proportion represented higher quality of care: 1) Proportion of patients with a negative malaria test result who were prescribed an antimalarial, and 2) Proportion of patients with a positive malaria test result who were prescribed an antibiotic. In five out of the nine indicators arm B performed better than arm A at baseline, with the absolute advantage ranging from 3% to 17%. For the remaining four indicators at baseline, arm A performed better than arm B with an absolute advantage ranging from 1% to 7%.

### Outcomes and Estimation

#### Effect of delayed OSS intervention in arm B

**Fig 3:**
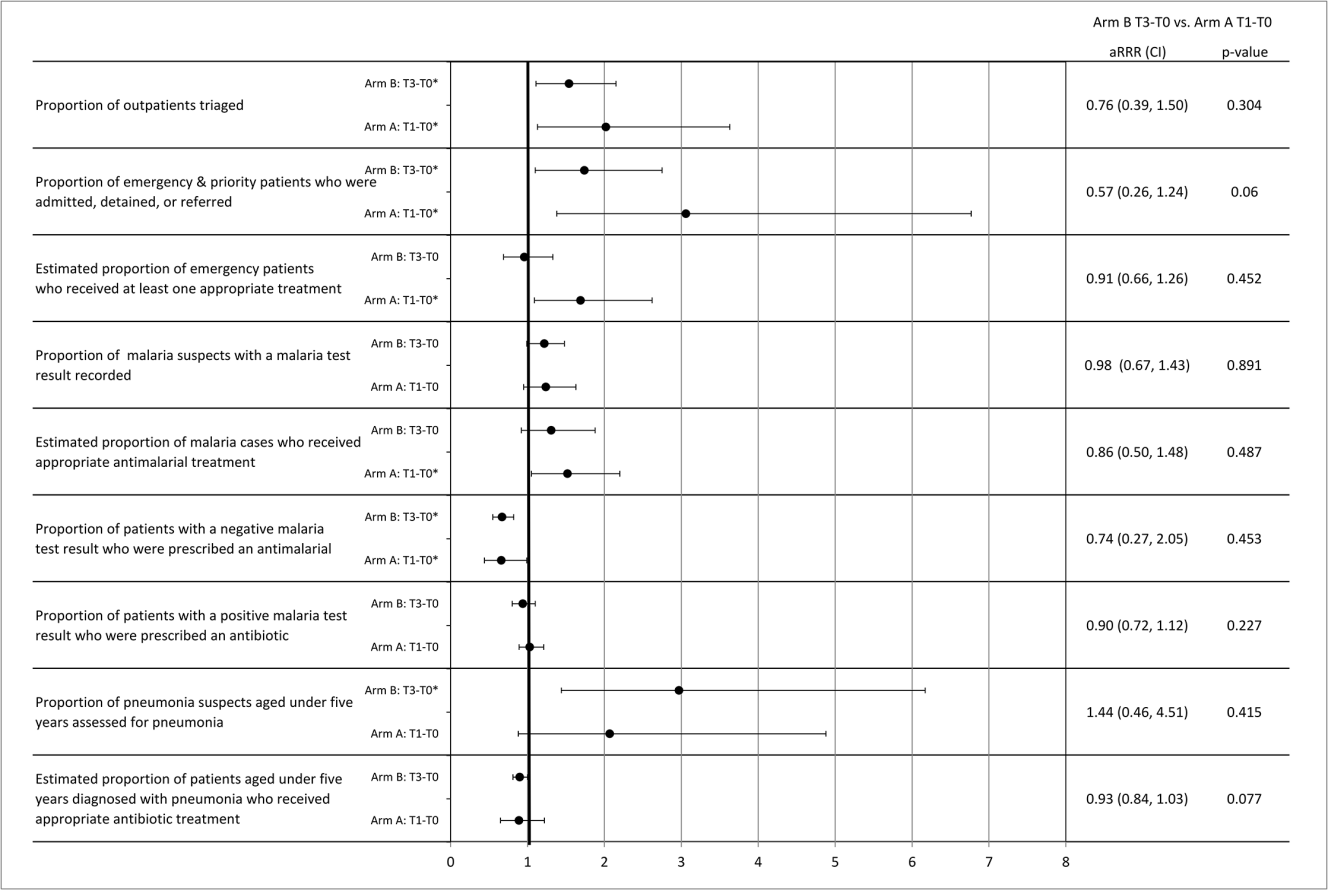
Adjusted relative risk ratios comparing the effect of training and OSS on study arms. *p<0.01. Time 0 (T0) is baseline for both arms. Time 1 (T1) for arm A and time 3 (T3)for arm B are the time periods after both the Integrated Management of Infectious Disease (IMID) training and the on-site support (OSS) educational outreach interventions. Arm B T3-T0 vs. arm A T1-T0 compares the change in the arm B to the change in arm A before and after the two interventions.

The results of three tests are reported in Fig 3: 1) whether or not performance improved in the delayed OSS arm, arm B, between time 0 and time 3 is reported as the relative risk of the indicator in time 3 compared to time 0, 2) whether or not performance improved in the immediate OSS arm, arm A, between time 0 and time 1 (which has previously been reported in Weaver et al. [13] with minor differences due the additional time periods), and 3) whether or not the magnitude of the improvement in the delayed OSS arm B was less than arm A is reported as the ratio of relative risk in arm B time 3 to time 0 to the relative risk in arm A time 1 to time 0.

In the multivariate analysis there was a significant combined effect of training and delayed OSS in four out of nine indicators in arm B when comparing time 0 and time 3, the same as the combined effect for training and immediate OSS in arm A when comparing time 0 and time 1 (Fig 3). Three of the four indicators that showed improvement were the same: outpatients triaged (arm B: 44% vs. 87%, aRR = 1.54, 99% CI = 1.11, 2.15; arm A: 27% vs. 86%, aRR = 2.02, 99% CI = 1.13, 3.63), emergency and priority patients who were admitted, detained, or referred (arm B: 16% vs. 31%, aRR = 1.74, 99% CI = 1.10, 2.75; arm A: 11% vs. 37%, aRR = 3.06, 99% CI = 1.38, 6.77), and patients with a negative malaria test result prescribed an antimalarial (arm B: 53% vs. 34%, aRR = 0.67, 99% CI = 0.55, 0.82; arm A: 46% vs. 30%, aRR = 0.66, 99% CI = 0.44,0.99). The proportion of pneumonia suspects aged under five years assessed for pneumonia showed significant improvement in arm B (6% vs. 27%, aRR = 2.97, 99% CI = 1.44, 6.17) but not arm A (3% vs. 16%, aRR = 2.07, 99% CI = 0.88, 4.88). The estimated proportion of malaria cases who received appropriate antimalarial treatment improved significantly in arm A (44% vs. 72%, aRR = 1.52, 99% CI = 1.05, 2.20) but not arm B (55% vs. 75%, aRR = 1.31, 99% CI = 0.92, 1.88).

Difference-in-difference in improvements in arm B from baseline to after training and delayed OSS compared to arm A were sometimes large, but not statistically significantly for any of the nine indicators (Fig 3).

#### Effect of OSS extension in arm A

**Fig 4:**
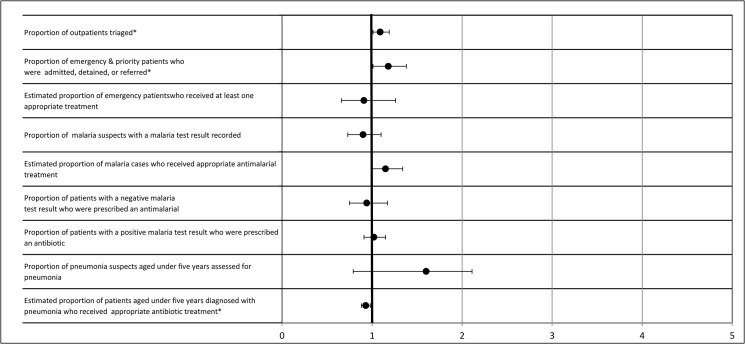
Adjusted relative risk ratios assessing the effect of the extended OSS period on Arm A. *p<0.01. This figure measures the additional effect of extended OSS in arm A by compares Time 3 in this arm, after the Integrated Management of Infectious Disease (IMID) training, the on-site support (OSS) educational outreach, and extended OSS to time 1, the period after IMID training and OSS.

The relative risk of facility performance during extended OSS (time 3) was compared to performance during infectious disease training and OSS (time 1) (Fig 4). In the regression analysis, two emergency triage, assessment, and treatment indicators showed significant additional improvement: 1) outpatients triaged (Arm A: 86% vs. 95%, aRR = 1.09, 99% CI = 1.01, 1.19) and 2) emergency and priority patients who were admitted, detained, or referred (Arm A: 37% vs. 45%, aRR = 1.22, 99% CI = 1.01, 1.38) (Fig 4). The estimated proportion of patients aged under five years diagnosed with pneumonia who received an appropriate antibiotic treatment significantly declined during time 3 (59% vs. 53%, aRR: 0.93; 99% CI = 0.88, 0.98). The difference-in-difference across the other six indicators were small and not statistically significantly different from the training and immediate OSS period.

## Discussion

Despite the nine months between the infectious disease training program and implementation of on-site support, the combined effect of these interventions was similar to the sequential implementation, with both the delayed and immediate on-site support associated with improvement in four of nine indicators. While the magnitude of change tended to be smaller with delayed than with immediate on-site support, this difference was not significant for any of the nine indicators. Thus, educational outreach, in this case coupled with continuous quality improvement activities, held up to nine months after an initial training can still lead to improvement in health facility indicators.

The on-site support extension period showed significant improvement for two indicators (outpatients triaged and emergency and priority patients who were admitted, detained, or referred), a significant decrease in one indicator (patients aged under five years who received appropriate antibiotic treatment) and no significant change for the remaining six indicators. A reduced level of effort for on-site support, conducting visits every other month rather than every month, had heterogeneous effects during six months after the trial, but was effective in maintaining improvements made during the more intensive time period. This corroborates data from a pre/post study on malaria case management, in which on-going monthly or bi-monthly site visits led to sustained improvements in clinical performance up to one year after training [30]. Facility performance indicators at these sites continued to improve after the first year, reaching nearly perfect performance for malaria diagnostic testing and appropriate treatment after four years of implementation[31].

This study adds new information about the effect of timing, duration, and frequency of educational outreach activities on facility performance. The delayed on-site support arm provided a rare opportunity to rigorously assess the timing of on-site support in relation to training. In most studies published on training and on-site support interventions, the on-site support immediately follows training. However, on a larger scale such well-timed interventions are not always possible, as program managers face competing priorities when scheduling interventions. Based on the findings presented, training program managers should be encouraged that they can implement an educational outreach intervention up to nine months after training with statistically significant improvements in performance. These findings may also be useful for designing and implementing inter-professional and community-based education programs for pre-service health professionals, such as the Medical Education Partnership Initiative (MEPI) [32,33].

While the majority of indicators showed large improvements over time, five out of the nine indicators were still below 60% and one reverse coded indicator, patients with a positive malaria test who were prescribed an antibiotic remained above 40% at time 3, after several months of OSS visits. This is consistent with findings from other studies, in which post-intervention practice was often less than 50% of desired performance [11]. The median level of improvement in our study was 16% (range -1% to 59%), consistent with the review of educational outreach visits, which reported a median relative improvement in performance indicators of 21.0% (interquartile range 11% to 41%) [11]. The majority of studies in this review were conducted in high resource settings and these results were achieved after one or two visits. Given the lower level of pre-service training and lack of infrastructure, staffing and support in limited resource settings, a longer duration and more concentrated effort may be required to achieve an effect similar to those found in high resource settings [13].

We may have had unrealistic expectations for improvements in the nine facility performance indicators presented here and 23 indicators in Weaver et al [13], even after including the additional six months of intervention in the extended OSS. Rather than trying to address a multitude of indicators at the same time, facilities could instead focus on one to four indicators until the desired level of performance is achieved before shifting the program’s focus to other performance indicators.

A program review by the HealthCare Improvement Project assessed 27 collaborative quality improvement projects across 12 countries [34]. In pre/post analyses, these projects demonstrated an average of 50% improvement after one year and projects focused on one to seven quality of care indicators (an average of 3.75), usually within one focus area (i.e. maternal health, HIV, TB, malaria). The Joint Uganda Malaria Program (JUMP) program, on which the IDCAP program was modeled, combined training with a malaria surveillance program that included on-site visits every one to two months. Selected sites improved two key malaria indicators to above 90% after four years of implementation [31].

A longer intervention duration, which slowly integrates multiple diseases, may be a more effective method for improving quality of care. Yet, even the modest facility performance improvements observed during the IDCAP intervention may translate into significant changes in health outcomes at the population level. An epidemiological model of the combined effects of IMID training and on-site support in Phase 1 showed reduced malaria prevalence by over 16% (Ssebuliba et al., unpublished manuscript).

In Uganda, donor-funded NGOs are supporting the Ugandan Ministry of Health to conduct supervision, with the majority of these programs focusing on vertical programs, such as HIV, TB or malaria. Integrating OSS to cover multiple diseases and sequencing the focus of visits could reduce redundancies in disease-specific support visits, such as assessing infrastructure and stock issues, and reducing the additional staff time and transport expenses related with supporting multiple single-disease interventions. Currently, the Ugandan Ministry of Health is encouraging this type of integrated supervision across disease areas (Mbonye, personal communication). The design of IDCAP was based on the JUMP model, which has been successfully scaled in Uganda [27]. Based on the large effects of IMID and OSS on several indicators found in this study, further operational research is needed to determine whether a phased approach focused on improving a small set of related indicators and building in additional disease areas over a longer period of time would produce effect on an integrated set of facility performance indicators.

### Limitations

Uptake of the intervention in the delayed OSS was lower than in the immediate on-site support, primarily due to the inclusion of a greater number of hospitals. This may have led to the smaller magnitude of change observed in the delayed OSS arm compared to the immediate on-site support. The sample size for this study was designed to detect a difference between arms at a five percent level of significance and may not be sufficient to detect a difference-in-difference at the one percent level of significance comparing two arms before and during the training and on-site support interventions. The accuracy of the data were not validated. It is possible that patients meeting the denominator definitions (i.e. emergency patients, malaria suspects, pneumonia suspects) were present but were not recorded, which would lead to an under reporting of cases for these indicators. The “post” analysis period was the period during the implementation of on-site support, rather than after, which may have led to an underestimation of the effect of the intervention. The pre/post analysis components, to measure the effect of delayed on-site support on arm B and the effect of the on-site support extension period on arm A, did not control for other changes at the sites over the course of the intervention. Also, in this study we did not test maintenance of facility performance after discontinuation of OSS. Given that indicators in arm A showed no significant decline during January to February 2011, the two months when no OSS took place, it is possible that the facility performance at these sites would have been maintained without the additional bi-monthly OSS. Further research is needed to determine whether facility performance can be maintained in the absence of on-going educational outreach.

### Generalizability

Eligibility criteria for IDCAP focused on health centers IV and comparable small hospitals in Uganda that met the inclusion criteria, thus these results would only be generalizable to these health facilities in Uganda. However, to the extent that these health facilities are similar to other primary care facilities throughout sub-Saharan Africa these results may inform the design and implementation educational outreach in other settings.

## Conclusions

Educational outreach held up to nine months after training had statistically significant effects on facility performance. Bi-monthly educational outreach maintained gains made in facility performance, but incremental improvements were heterogeneous.

## Supporting Information

S1 Table CONSORT Checklist. A table which presents the detailed CONSORT checklist, which includes the CONSORT extension for cluster trials.
(PDF)

S1 Fig Proportion of patients managed appropriately for all nine indicators by arm and time. Raw proportions and sample sizes for each of the nine facility performance indicators presented by arm and time period.
(PDF)
