# Supplementary material for: Effect of Educational Outreach Timing and Duration on Facility Performance for Infectious Disease Care in Uganda: A Trial with Pre-Post and Cluster Randomized Controlled Components
Source: PLoS One. 2015 Sep 9;10(9):e0136966. doi: 10.1371/journal.pone.0136966 (PMC4564214; doi:10.1371/journal.pone.0136966)
Supplement: S1 Table — A table which presents the detailed CONSORT checklist, which includes the CONSORT extension for cluster trials. (PDF) [file pone.0136966.s001.pdf]

S1 Table. CONSORT checklist for cluster randomised trials

PAPER SECTION and topic	Item	Descriptor	Reported on Page No.
TITLE & ABSTRACT			
	1a*	Identification as a randomised trial in the title	Title
	1b	How participants were allocated to interventions (e.g., “random allocation”, “randomised”, or “randomly assigned”), *specifying that allocation was based on clusters*	Abstract
INTRODUCTION			
Background & Objectives	2a	Scientific background and explanation of rationale, *including the rationale for using a cluster design.*	Introduction, Study design, Naikoba et al. [6]
	2b	Specific objectives or hypotheses, *Whether objectives pertain to the cluster level, the individual participant level or both*	Background, Study design
METHODS			
Trial Design	3a	Description of trial design (such as parallel, factorial) including allocation ratio	Study design
	3b	Important changes to methods after trial commencement (such as eligibility criteria), with reasons	Interventions
Participants	4a	Eligibility criteria for participants *and clusters* and the settings and locations where the data were collected.	Participants and eligibility, Miceli et al.[28], Naikoba et al.[6]
	4b	Settings and locations where the data were collected	Participants and eligibility, Naikoba et al.[6]
Interventions	5	Precise details of the interventions intended for each group, *whether they pertain to the individual level, the cluster level or both,* and how and when they were actually administered.	Interventions, Miceli et al.[28], Naikoba et al.[6]
Outcomes	6a	Report clearly defined primary and secondary outcome measures, *whether they pertain to the individual level, the cluster level or both*, and, when applicable, any methods used to enhance the quality of measurements (e.g., multiple observations, training of assessors).	Variable definitions and sources, Table 1
	6b	Any changes to trial outcomes after the trial commenced, with reasons	Variable definitions and sources, Weaver et al. [13]

PAPER SECTION and topic	Item	Descriptor	Reported on Page No.
Sample size	7a	How *total* sample size was determined *(including method of calculation, number of clusters, cluster size, a coefficient of intracluster correlation (ICC or k), and an indication of its uncertainty*) and, when applicable, explanation of any interim analyses and stopping rules.	Sample size, Naikoba et al.[6]
	7b	When applicable, explanation of any interim analyses and stopping guidelines	Not applicable
RANDOMIZATION			
Sequence generation	8a	Method used to generate the random allocation sequence,	Randomization Naikoba et al.[6], Weaver et. al.[13]
	8b	Type of randomisation; details of any restriction (such as blocking and block size) Details of stratification or matching if used	Randomization Naikoba et al.[6], Weaver et. al.[13]
Allocation concealment	9*	Method used to implement the random allocation sequence, *specifying that allocation was based on clusters rather than individuals and* clarifying whether the sequence was concealed until interventions were assigned.	Randomization Naikoba et al.[6]
Implemen-tation	10a	Who generated the allocation sequence, who enrolled participants, and who assigned participants to their groups.	Randomization Naikoba et al.[6]
	10b	Mechanism by which individual participants were included in clusters for the purposes of the trial (such as complete enumeration, random sampling)	Data collection
	10c	From whom consent was sought (representatives of the cluster, or individual cluster members, or both), and whether consent was sought before or after randomisation	Ethical considerations
Blinding	11a	Whether or not participants, those administering the interventions, and those assessing the outcomes were blinded to group assignment.	Randomization
	11b	If relevant, description of the similarity of interventions	Not applicable
Statistical methods	12a	Statistical methods used to compare groups for primary outcome(s) *indicating how clustering was taken into account*; methods for additional analyses, such as subgroup analyses and adjusted analyses.	Data analysis

PAPER SECTION and topic	Item	Descriptor	Reported on Page No.
	12b	Methods for additional analyses, such as subgroup analyses and adjusted analyses	Data analysis
RESULTS			
Participant flow	13a	Flow of *clusters and* individual participants through each stage (a diagram is strongly recommended). Specifically, for each group report the numbers of *clusters and* participants randomly assigned, receiving intended treatment, completing the study protocol, and analyzed for the primary outcome. Describe protocol deviations from study as planned, together with reasons.	Participant flow, Figure 1, Supplemental Figures 1-3
	13b	For each group, losses and exclusions after randomisation, together with reasons. For each group, losses and exclusions for both clusters and individual cluster members	Participant flow
Recruitment	14a	Dates defining the periods of recruitment and follow-up.	Recruitment
	14b	Why the trial ended or was stopped	Not applicable
Baseline data	15	Baseline information for each group *for the individual and cluster levels as applicable*	Weaver [13], Supplemental Figures 1-3
Numbers analyzed	16	Number of *clusters and* participants (denominator) in each group included in each analysis and whether the analysis was by "intention-to-treat". State the results in absolute numbers when feasible (e.g., 10/20, not 50%).	Participant flow, Figure 1, Supplemental Figures 1-3
Outcomes and Estimation	17a	For each primary and secondary outcome, a summary of results for each group measures *for the individual or cluster level as applicable*, and the estimated effect size and its precision (e.g., 95% confidence interval)	Outcomes and estimation, Figure 3, Figure 4
	17b	For binary outcomes, presentation of both absolute and relative effect sizes is recommended	Outcomes and estimation, Figure 3, Figure 4, Supplemental Figures 1-3
Ancillary analyses	18	Results of any other analyses performed, including subgroup analyses and adjusted analyses, distinguishing pre-specified from exploratory	Not applicable

PAPER SECTION and topic	Item	Descriptor	Reported on Page No.
Adverse events	19	All important adverse events or side effects in each intervention group.	Not applicable
DISCUSSION			
Interpretation	20	Interpretation consistent with results, balancing benefits and harms, and considering other relevant evidence	Discussion
Limitations	21	Trial limitations, addressing sources of potential bias, imprecision, and, if relevant, multiplicity of analyses	Limitations
Generalizability	22	Generalizability (external validity) *to individuals and/or clusters (as relevant)* of the trial findings	Generalizability
Overall evidence	23	General interpretation of the results in the context of current evidence.	Conclusion
OTHER INFORMATION			
Registration	24	Registration number and name of trial registry	Not applicable
Protocol	25	Where the full trial protocol can be accessed, if available	Study Design
Funding	26	Sources of funding and other support (such as supply of drugs), role of funders	Acknowledgemen ts
