# Supplementary material for: Effect of Educational Outreach Timing and Duration on Facility Performance for Infectious Disease Care in Uganda: A Trial with Pre-Post and Cluster Randomized Controlled Components
Source: PLoS One. 2015 Sep 9;10(9):e0136966. doi: 10.1371/journal.pone.0136966 (PMC4564214; doi:10.1371/journal.pone.0136966)
Supplement: S1 Fig — Raw proportions and sample sizes for each of the nine facility performance indicators presented by arm and time period. (PDF) [file pone.0136966.s002.pdf]

Supplemental Figure 1. Proportion of patients managed appropriately on three emergency triage, assessment, and treatment indicators, by arm and time

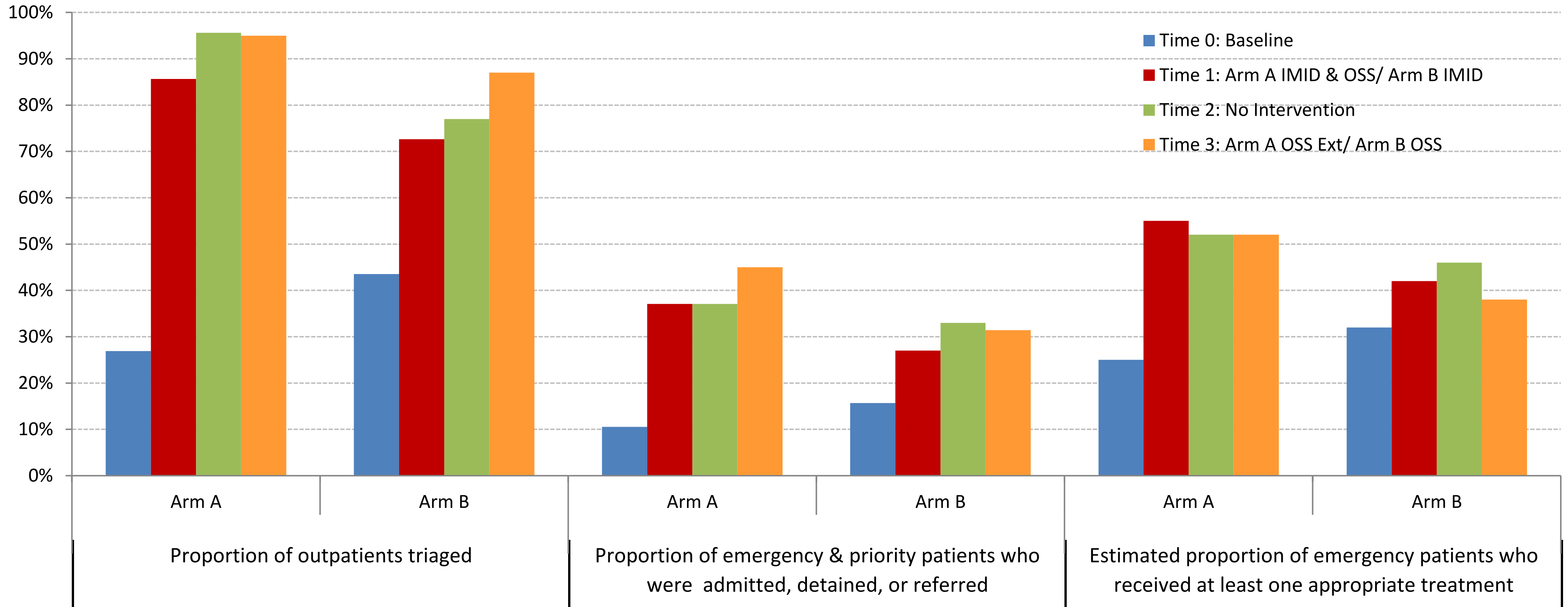

	Proportion of outpatients triaged		Proportion of emergency & priority patients who were admitted, detained, or referred		Estimated proportion of emergency patients who received at least one appropriate treatment	
	Arm A	Arm B	Arm A	Arm B	Arm A	Arm B
Time 0	94,812	195,371	6,337	23,663	2,677	9,960
Time 1	235,784	227,107	17,494	19,677	4,382	6,733
Time 2	48,465	55,127	3,397	3,838	616	1,239
Time 3	198,479	220,815	11,108	18,780	1,797	5,957

Numbers presented are the denominators for each of the indicators at each time period

Supplemental Figure 2. Proportion of patients managed appropriately on four malaria indicators, by arm and time

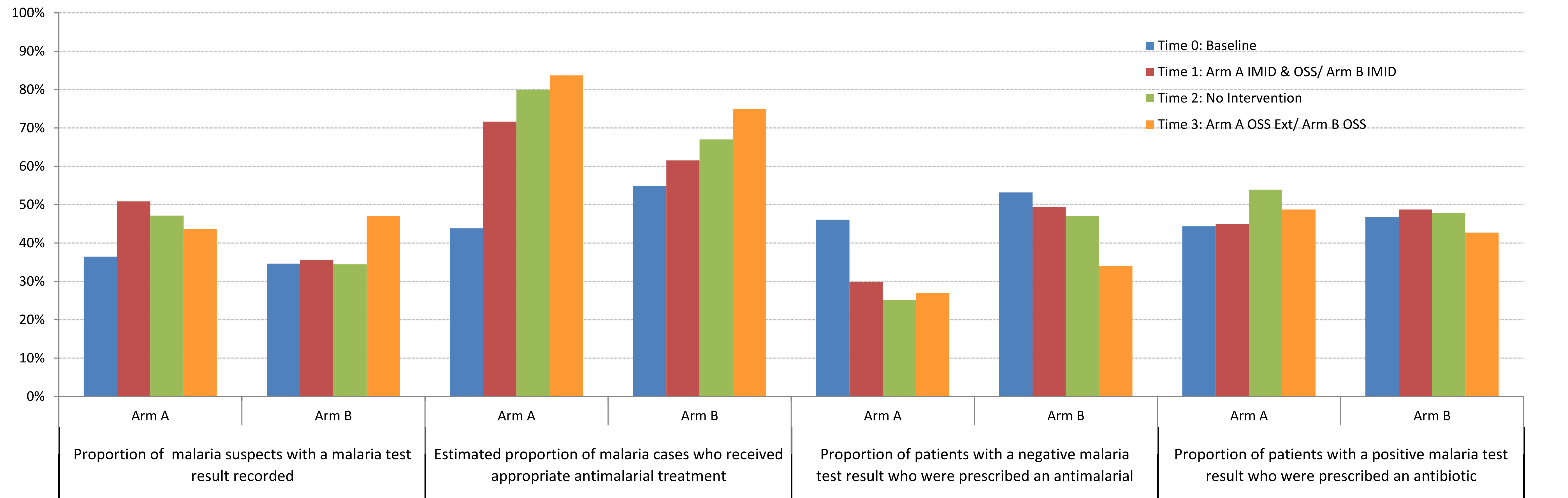

	Arm A	Arm B	Arm A	Arm B	Arm A	Arm B	Arm A	Arm B
Time 0	63,729	130,540	53,619	45,599	11,741	24,341	11498	20,867
Time 1	144,397	146,912	101,374	120,053	41,450	32,094	31996	20,293
Time 2	27,630	33,436	17,417	26,123	8,861	7,921	4217	3,438
Time 3	112,591	133,304	73,535	91,911	33,349	41,556	16287	21,335

Numbers presented are the denominators for each of the indicators at each time period

Supplemental Figure 3. Proportion of patients managed appropriately on two pneumonia indicators, by arm and time

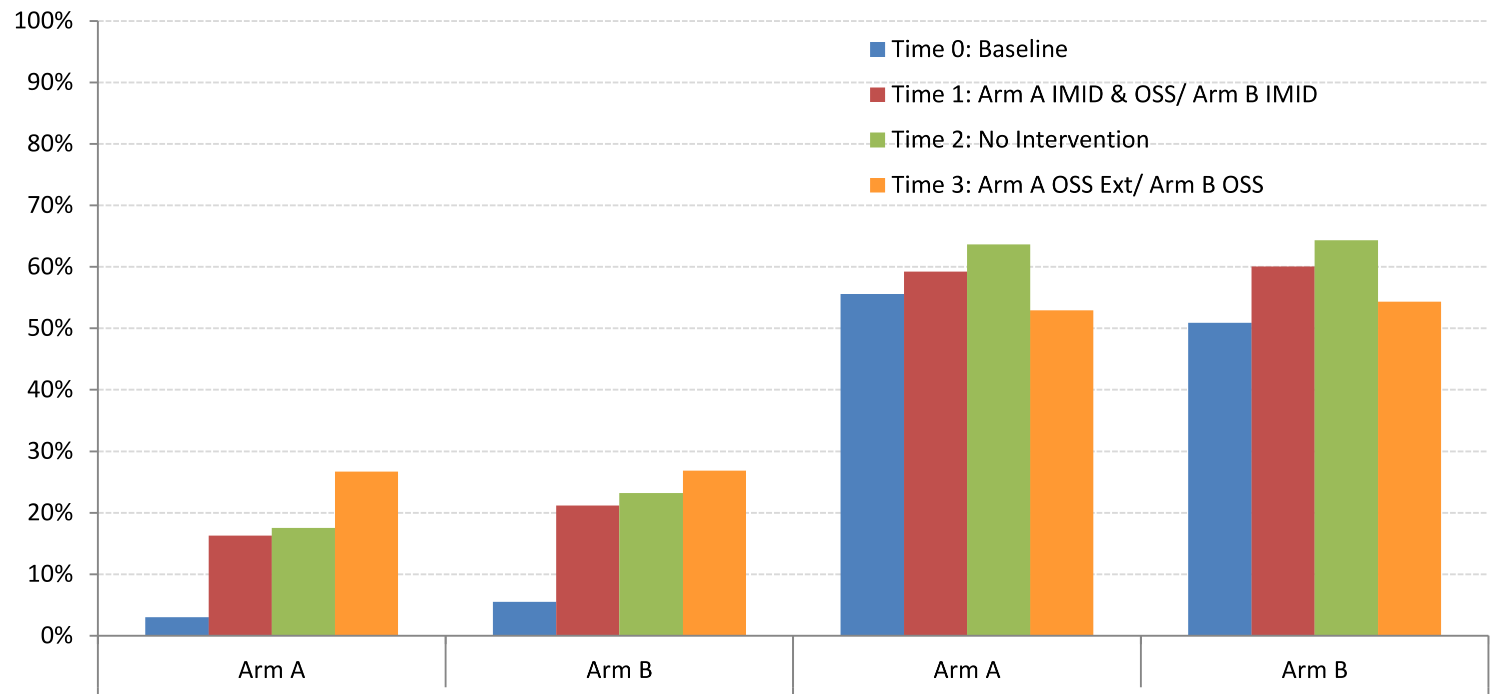

	Proportion of pneumonia suspects aged under five years assessed for pneumonia		Estimated proportion of patients aged under five years diagnosed with pneumonia who received appropriate antibiotic treatment	
	Arm A	Arm B	Arm A	Arm B
Time 0	13,378	31,358	1,623	5,257
Time 1	35,057	33,708	4,884	5,577
Time 2	6,852	7,258	935	1,217
Time 3	28,886	29,555	4,616	5,755

Numbers presented are the denominators for each of the indicators at each time period
